# Aminoacyl-tRNA synthetase interactions in SARS-CoV-2 infection

**DOI:** 10.1042/BST20230527

**Published:** 2023-12-18

**Authors:** Debjit Khan, Paul L. Fox

**Affiliations:** Department of Cardiovascular and Metabolic Sciences, Lerner Research Institute, Cleveland Clinic, Cleveland, Ohio, U.S.A.

**Keywords:** aminoacyl-tRNA synthetase, post-transcriptional region, RNA virus, SARS-COV-2, UTR

## Abstract

Aminoacyl-tRNA synthetases (aaRSs) are ancient enzymes that serve a foundational role in the efficient and accurate translation of genetic information from messenger RNA to proteins. These proteins play critical, non-canonical functions in a multitude of cellular processes. Multiple viruses are known to hijack the functions of aaRSs for proviral outcomes, while cells modify antiviral responses through non-canonical functions of certain synthetases. Recent findings have revealed that severe acute respiratory syndrome coronavirus 2 (SARS-CoV-2), the etiological agent of coronaviral disease 19 (COVID-19), utilizes canonical and non-canonical functions of aaRSs, establishing a complex interplay of viral proteins, cellular factors and host aaRSs. In a striking example, an unconventional multi-aaRS complex consisting of glutamyl-prolyl-, lysyl-, arginyl- and methionyl-tRNA synthetases interact with a previously unknown RNA-element in the 3′-end of SARS-CoV-2 genomic and subgenomic RNAs. This review aims to highlight the aaRS-SARS-CoV-2 interactions identified to date, with possible implications for the biology of host aaRSs in SARS-CoV-2 infection.

## An introduction to aminoacyl-tRNA synthetases

Aminoacyl-tRNA synthetases (aaRSs) are essential cellular enzymes that provide aminoacylated tRNAs as substrates to ribosomes as genetic information is decoded from messenger RNA to proteins. AaRSs catalyze the aminoacylation reaction in two steps: first, activation of an amino acid (aa) by a condensation reaction with ATP accompanied by release of pyrophosphate followed by esterification of the amino acid with a tRNA, and release of AMP [1–3]. AaRS-catalyzed tRNA-charging serves two purposes — (i) the amino acid remains energetically activated, i.e. its subsequent condensation with another amino acid in a peptide chain does not require additional energy input, and (ii) more importantly, provides the information required to recognize the mRNA template that determines sequence specificity. Each aaRS specifically recognizes its corresponding amino acid and cognate tRNA. Specific structural features and nucleotide sequences in tRNA isoacceptor molecules (tRNA^aa^), the operational RNA code [4,5], as well as identity of the amino acids (aa) guide precise aminoacylation to generate the aa-tRNA^aa^. In addition to anticodon binding and catalysis, several aaRSs have *cis*-editing domains that recognize and correct mischarging on non-cognate tRNAs [6]. Proteins that lack a tRNA-charging function but otherwise resemble aaRS editing domains can proofread mischarged aminoacylated tRNAs by *trans*-editing [7]. There are 20 aaRSs for the 20 amino acids enshrined in the genetic code. With some degree of evolutionary mosaicism selenocysteine (Sec), the 21st proteinogenic amino acid, is present in the three domains of life (eubacteria, archaea, and eukarya), and is generated from serine on seryl-tRNA^Sec^, after tRNA^Sec^ is charged with seryl-tRNA synthetase [8,9]. Certain methanogenic archaea possess an expanded genetic code that includes the 22nd proteinogenic amino acid pyrrolysine (Pyl) [10,11], that is charged to tRNA^Pyl^ by pyrrolysyl-tRNA synthetase [12].

The endosymbiotic origin of mitochondria in eukaryogenesis [13,14] resulted in two sets of operational RNA codes in the same cellular system: one from the host and another from the endosymbionts. Today, mitochondrial DNA encodes 22 tRNAs, while the cognate mitochondrial aaRSs (mt-aaRS) are nuclear DNA-encoded. To distinguish between human cytosolic and mt-aaRS, the standard nomenclature uses 1 or 2 as a suffix for any aaRS protein, e.g. seryl-tRNA synthetase 1 or SARS1 is cytosolic while SARS2 is mt-SARS. As exceptions: (i) human *GARS1* gene encodes glycyl-tRNA synthetase that functions in both cytosol and mitochondria [15], (ii) human KARS2 protein is an isoform of KARS1 protein produced from the *KARS1* gene [16], (iii) mt-QARS is absent from mammalian genomes; EARS2 misacylates mt-tRNA^Gln^ and glutamine is synthesized on Glu-mt-tRNA^Gln^ post-aminoacylation by the hGatCAB amidotransferase [17], and (iv) EPRS1 in complex eukaryotes is a unique, bifunctional aaRS that resides in the cytosol and consists of an N-terminus GluRS domain covalently joined by a linker domain to a C-terminus ProRS domain [18].

## Aminoacyl-tRNA synthetases in virus infection

Viruses are obligate, intracellular parasites that carry minimal genetic information compared with its hosts, and thus depend on usurping host cellular resources. Most viruses do not encumber their genomes with aaRSs, instead they rely on host aaRSs and other host proteins and complexes that enable mRNA translation, e.g. ribosomes. Exceptions include several giant DNA viruses with protozoan hosts that encode a single aaRS or a small complement of aaRSs, e.g. *Cafeteria roenbergensis* virus (isoleucyl-tRNA synthetase) [19], Pandoraviruses (tyrosyl and tryptophanyl-tRNA synthetases) [20], Mimiviruses (tyrosyl, methionyl, arginyl and cysteinyl-tRNA synthetases) [21], Moumouviruses (tyrosyl, methionyl, arginyl, cysteinyl and isoleucyl-tRNA synthetases) [22] and *Megavirus chilensi*s (tyrosyl, methionyl, arginyl, cysteinyl, isoleucyl, tryptophanyl and asparginyl-tRNA synthetases) [23], while Tupanviruses encode all 20 aaRSs [24]. Host aaRS aminoacylation activity can be hijacked by 3′-localized tRNA-like structures (TLS) that control replication of plant RNA viruses [25,26]. The 3′-TLS in ∼30 plant viruses are aminoacylated by YARS1 (e.g. brome mosaic virus, BMV), HARS1 (e.g. tobacco mosaic virus), or VARS1 (e.g. turnip yellow mosaic virus), and tRNA mimicry is essential for plant virus replication and gene expression [27]. The BMV tRNA mimic undergoes a conformational rearrangement, binding YARS1 in a structural form that differs dramatically from tRNA [28], exemplifying the dynamics of viral RNA structures in binding host machinery. In a second case of tRNA mimicry, domain V of the internal ribosome entry site (IRES) in poliovirus genome resembles tRNA^Gly^, and binds GARS1 to activate translation–initiation [29].

**Figure 1.**
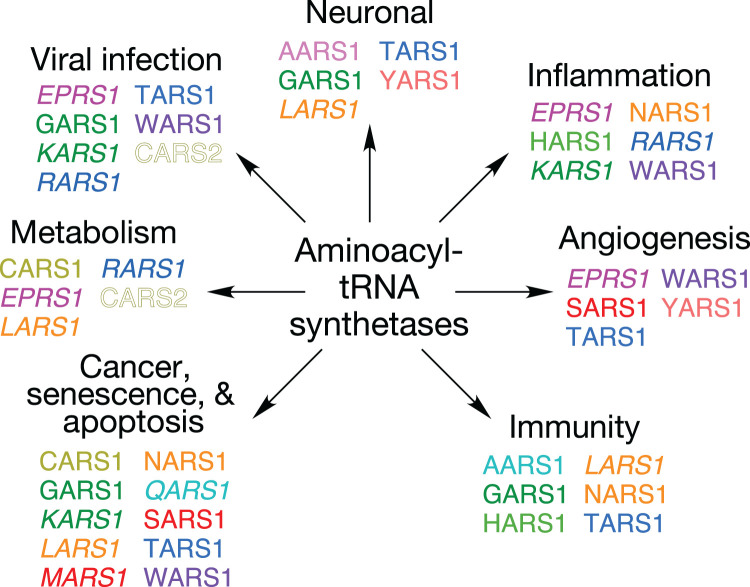
Non-canonical functions of aminoacyl-tRNA synthetases. Aminoacyl-tRNA synthetases are color-coded to highlight multiple functions of same protein. MSC-resident (italics), non-MSC cytoplasmic (plain font), and mitochondrial (outline font) synthetases are indicated.

Many mammalian aaRSs exhibit non-canonical functions distinct from their ancient, primary function of tRNA aminoacylation (Figure 1). These moonlighting, or ‘ex-translational', activities generally depend on domains appended in evolution [30]. These functions are regulated by post-translational modifications [31–36] or by targeted cleavage [37–40]. These modified aaRS can act as cytokines, apoptosis and angiogenesis regulators, and non-enzymatic regulators of translation, with the potential to integrate genetic and environmental responses [41–43]. In mammalian cells, nine of the 20 cytoplasmic aaRS functions (in 8 proteins since EPRS1 is bifunctional) and 3 auxiliary proteins, AIMPs (aminoacyl-tRNA synthetase interacting multifunctional proteins) 1, 2, and 3, reside in a multi-tRNA synthetase complex (MSC), of uncertain function. The observation of MSC binding to ribosomes has led to the hypothesis that MSC ‘channeling' of charged tRNAs to ribosomes improves mRNA translation efficiency [44]. This concept is challenged by reports that global translation is not reduced when the majority of EPRS1, or all of RARS1 and QARS1, are excluded from the MSC [41,45]. Alternatively, the MSC might sequester aaRSs to reduce injurious cell activities of the free proteins, while permitting cue-dependent release of specific aaRSs for non-canonical functions [41,46].

Importantly, several aaRSs exhibit critical non-canonical, host-viral interactions, and consequent activities, as follows: infection-dependent release of EPRS1 from the MSC sequesters poly(rC)-binding protein 2 (PCBP2) and protects MAVS, an antiviral mitochondrial signaling molecule, from PCBP2-mediated ubiquitination in influenza A virus-infected cells [47]. EPRS1 and RARS1 bind an RNA element in porcine transmissible gastroenteritis coronavirus (TGEV), thereby facilitating innate immune evasion of viral RNA genome [48]. Human immunodeficiency virus type 1 (HIV-1) virions are packaged with tRNA^Lys3^ by KARS1, that is incorporated into the virus following regulated release from the MSC [49,50]. The HIV-1 genome contains a tRNA-like element (TLE) that acts as molecular mimic of tRNA^Lys^ and aids tRNA^Lys3^ primer annealing by recruitment of KARS1 [51]. HIV-1 gag protein forms a stable complex with the MSC through a tRNA-dependent interaction with the EPRS1 linker domain, without specificity of the tRNA utilized [52].

Non-MSC, cytoplasmic aaRSs and mitochondrial KARS2 contribute to viral life cycles [53]. Similarly, WARS1 is an interferon (IFN)-γ-inducible mediator of enterovirus, e.g. EV-A71, cell entry, and a cell type-specific restriction factor [54]. In contrast, immune cells infected with vesicular stomatitis virus (VSV) or herpes simplex virus (HSV) secrete WARS1 that functions as an antiviral cytokine, promoting the production of inflammatory cytokines and type-I IFNs to suppress virus replication [55]. Likewise, TARS1 is secreted from vascular endothelial cells in response to tumor necrosis factor (TNF)-α and sculpts a T-helper-1 response for clearing H1N1 influenza A virus infection [56].

## SARS-CoV-2 and the COVID-19 pandemic

During the last 4 years, coronaviral disease-19 (COVID-19) has upended lives with levels of mortality and morbidity unprece­dented in recent history [57–60]. COVID-19 is the first documented coronavirus pandemic in human history [61], instigating widespread public discourse on pharmaceutical and non-pharmaceutical interventions [62–64]. Severe acute respiratory syndrome coronavirus 2 (SARS-CoV-2) is the causative agent of COVID-19, and the seventh coronavirus known to cause a human disease [65,66]. SARS-CoV-2 is an enveloped betacoronavirus with positive, single-stranded genomic RNA, closely related to SARS-CoV-1 and to bat sarbecoviruses [67]. Serious cases of COVID-19 exhibit acute respiratory distress syndrome (ARDS) [68] and can be lethal, particularly if exacerbated by co-morbidities [69]. Extended ramifications of the disease manifest as ‘long COVID' that remains incompletely understood or even phenotyped [70–73]. Possibly, waves of SARS-CoV-2 infection in the COVID-19 pandemic has reduced genetic diversity of other respiratory viruses, such as respiratory syncytial virus (RSV) and influenza A virus, through bottleneck effects, thereby re-shaping future disease outbreaks [74]. Based on adaptive functions of other viruses, it is not surprising that SARS-CoV-2 took advantage of host systems, including piggybacking on cellular aaRSs.

## Mitochondrial aaRSs as mediators of SARS-CoV-2 infection

To evaluate the roles of aaRSs on SARS-CoV-2 infection and pathogenesis, a meta-analysis summarized findings from multiple datasets [75]. A graded cell survival analysis was done by genome-wide CRISPR screening of Vero-E6 cells following infection with SARS-CoV-2 and other coronaviruses (CoVs) [76]. Disruption of 14 mitochondrial aaRSs (AARS2, DARS2, EARS2, FARS2, HARS2, LARS2, MARS2, NARS2, PARS2, RARS2, SARS2, TARS2, VARS2, and YARS2) sensitized cells to cell death following infection by SARS-CoV-2, suggesting antiviral activity of the aaRSs. PARS2 and EARS2 appear to exert pan-coronavirus antiviral activity as the screen showed sensitization to cell death upon infections with SARS-CoV-2, HKU5-SARSCoV-1-S, and MERS-CoV. EARS2 was further implicated in cellular entry of SARS-CoV-2. Mitochondrial aaRSs appeared to be more effective than cytosolic aaRSs as antiviral effectors/modulators following SARS-CoV-2 infection [75].

SARS-CoV-2 initiates a global shutdown of splicing, mRNA nuclear export, and translation [77]. Consistent with this repression, down-regulation of mRNAs encoding cytosolic and mitochondrial aaRSs was observed in transcriptomic studies of bronchoalveolar lavage fluid (BALF) [78] and post-mortem lung tissues [79] from COVID-19 patients, and from SARS-CoV-2-infected A549-hACE2 (human angiotensin-converting enzyme) [79] and human bronchial epithelial (NHBE) cells [80]. Likewise, low levels of many cytosolic aaRS proteins were observed in proteomic analyses of BALF [81], peripheral blood mononuclear cells (PBMCs) [82], and liver cells [83] from COVID-19 patients, and from SARS-CoV-2-infected human Caco-2 cells [84]. Many mt-aaRS proteins are expressed at high levels in liver cells from COVID-19 patients, but the levels of mt-aaRS mRNAs were not reported. The phenomenon seems to be generalizable as multiple mitochondrial ribosomal proteins are also expressed at high levels in liver cells from COVID-19 patients, likely reflecting increased mitochondrial translation. Phosphoproteomic analysis of Vero-E6 cells infected with SARS-CoV-2 revealed phosphorylation of CARS1, TARS2, and HARS2 [85].

Meta-analysis of proteomic studies sub-divided coronaviral protein-interacting aaRSs into three classes, namely, down-regulated mt-aaRSs, down-regulated cyto-aaRSs, and up-regulated mt-aaRSs [75]. Network analysis of aaRSs implicated EARS2, IARS1, IARS2, and TARS1 as mediators between first responders, i.e. SARS-CoV-2-interacting proteins and late-stage effectors. SARS-CoV-2 membrane protein (M) interacts with TARS2 in HEK293T cells [75], but the physiological significance is unknown. While the analysis implicates mt-aaRSs, particularly HARS2, EARS2, and TARS2 in SARS-COV-2 infection severity, the consequences of differential aaRS expression remain unclear. Whether such changes benefit the virus or the host cell is unclear, but would differentially implicate either proviral or antiviral outcomes. Multiple recent studies [86–108] have expanded the aaRS-interaction datasets beyond those in this meta-analysis; interactions of cellular aaRSs and SARS-CoV-2 proteins based on BioGrid repository are enumerated as in Table 1.

**Table 1:** **Putative interactions between host aminoacyl-tRNA synthetases and SARS-CoV-2 proteins curated from the BioGrid database** [143]

AaRS	SARS-CoV-2 non-structural proteins	SARS-CoV-2 structural proteins	References	
AARS1	NSP10, NSP11, NSP16	S, ORF9B, ORF10	[82, 87–89, 106]
CARS1	-	-	
DARS1	NSP4, NSP5, NSP6, NSP12, NSP13, NSP14	S, ORF3A, ORF3B, E, M, ORF6, ORF7A, ORF7B, ORF8	[90, 104, 106]
EPRS1	NSP5, NSP12	ORF8, ORF14 (ORF9C)	[90, 102–104]
FARSA	NSP2	ORF10	[88, 92]
FARSB	-	ORF8, N	[103]
GARS	-	-	
HARS1	-	-	
IARS1	NSP5	-	[90]
KARS1/ KARS2	NSP5, NSP7, NSP15	ORF3A, ORF3B, E, M, ORF6, ORF7A, ORF7B, ORF8, N, ORF9B, ORF10	[87, 90, 91, 105, 106]
LARS1	-	-	
MARS1	NSP1, NSP5	ORF14 (ORF9C)	[90, 94, 102]
NARS1	NSP2, NSP4, NSP6	ORF3A, M, ORF7B, ORF10	[87, 106]
QARS1	NSP4	S, ORF6, ORF7B, ORF8, ORF9B, ORF10	[87, 106]
RARS1	-	N	[91]
SARS1	NSP15	N	[87, 105]
TARS1	NSP2, NSP4	S, ORF7B, ORF8, N, ORF10	[87, 93]
VARS1	NSP2	S, ORF8, ORF10	[88, 103]
WARS1	NSP9, NSP15	N	[93, 103]
YARS1	NSP4, NSP5	S, E, M, ORF6, ORF8, N, ORF9B, ORF10	[87, 90, 91, 106]
AARS2	NSP5, NSP9	ORF7B, ORF9B	[90, 95, 105, 106]
CARS2	-	ORF9B	[106]
DARS2	NSP4, NSP5	M, ORF9B	[90, 106]
EARS2	NSP4	S, ORF7A, ORF7B, ORF9B, ORF14 (ORF9C)	[105, 106]
FARS2	-	-	
HARS2	-	M, ORF9B	[86, 106]
IARS2	NSP2, NSP3, NSP5, NSP6, NSP9, NSP13, NSP14, NSP16	S, ORF3A, ORF14 (ORF9C), ORF10	[87–89, 102]
LARS2	-	-	
MARS2	NSP1, NSP14	-	[93]
NARS2	NSP8	M	[86, 96, 97]
PARS2	-	-	
RARS2	NSP6, NSP8	ORF9B	[82, 100, 106, 107]
SARS2	NSP8	-	[87]
TARS2	NSP7, NSP10, NSP12, NSP13	M, ORF9B	[82, 85, 96, 97, 100, 104, 105, 108]
VARS2	-	ORF9B	[106]
WARS2	-	-	
YARS2	NSP1, NSP4	-	[87]
			
Non-AARS, MSC-resident proteins			
AIMP1	NSP5	ORF3B, ORF6, N	[87, 90, 98, 103]
AIMP2	-	ORF8, N	[103]
AIMP3	NSP5, NSP13	ORF3A, ORF9B, ORF10	[87, 99]

All references listed in text.

## A ‘post-canonical' protective activity of CARS2

Recently, protective functions of cysteine hydropersulfides and cysteine supersulfides were identified in lung diseases, including viral airway infections such as influenza and COVID-19 [109]. Cysteine hydropersulfides (CysSSH) have been implicated in cellular redox protection. Mitochondrial cysteinyl-tRNA synthetase (CARS2) exhibits cysteine persulfide synthase (CPERS) activity *in vivo* [110]. CARS2 generates CysS–(S)*_n_*–H from cysteine post-aminoacylation through CPERS activity, forming CysS–(S)*_n_*–tRNA^Cys^ from Cys-tRNA^Cys^, and catalyzing co-translational protein polysulfidation. Importantly, CPERS-mediated polysulfidation occurs on aminoacylated tRNA, contingent on and following the canonical function of CARS2, thereby revealing a ‘post-canonical' aaRS function. CARS2-derived cysteine hydropersulfides and cysteine supersulfides sustain mitochondrial biogenesis and activity of the electron transport chain [110]. Also, CysSSH is released from the mitochondria into the cytoplasm for production of CysS–(S)*_n_*–H and polysulfidation in extra-mitochondrial compartments. Increased SARS-CoV-2 yield was observed in *CARS2* knockdown VeroE6/TMPRSS2 cells. *Cars2*^AINK^ mutant mice possess undiminished cysteinyl-tRNA synthetase activity, but exhibit reduced CPERS activity and low supersulfide production [109]. Homozygous *Cars2*^AINK/AINK^ mice are embryonic lethal indicating a developmental role of supersulfides. SARS-CoV-2 infection was assessed in *Cars2*^AINK^ mice crossed with ACE2-transgenic mice, and lethality was significantly exacerbated. Thus, CARS2 has a central role in innate defense functions of supersulfiides, protecting the lung and airways, as well as associated vasculature, against SARS-CoV-2 [109].

## ProRS and proline-rich proteins are potential therapeutic targets

Febrifugine, isolated from *Dichroa febrifuga* in the family Hydrangeaceae, is an alkaloid and active ingredient in a traditional Chinese medicinal herb, chángshān [111,112]. Recognized for its antiprotozoal activity, chángshān extract historically has been used as an anti-malarial treatment [113]. Halofuginone is a halogenated derivative of febrifugine that potently inhibits the differentiation of pro-inflammatory Th17 cells [114]. It binds the ProRS domain of human glutamyl-prolyl-tRNA synthetase (EPRS1), acting as a competitive inhibitor of prolyl-tRNA synthetase activity [115]. Halofuginone has been used in preclinical and clinical studies to treat fibrotic disease and to reduce hyperinflammation [116]. Screening a library of small-molecule antagonists of SARS-CoV-2 spike receptor-binding domain (RBD) interaction with extracellular heparan sulfate in Hep3B human hepatoma cells, halofuginone was identified as a potent hit [117]. The drug reduced RBD binding to Hep3B, Calu-3, and Caco-2 cells, and at low nanomolar amounts inhibited SARS-CoV-2 infection of Hep3B and air–liquid interface cultures of primary human bronchial epithelial cells. Spike binding to cells is heparan sulfate (HS)-dependent [118]. Halofuginone decreased cellular HS synthesis without altering HS-specific sulfation, and reduced expression of core heparan sulfate proteoglycans (HSPGs) [117]. These results established that pre-infection treatment with halofuginone inhibits SARS-CoV-2 entry in these models. Further, post-infection treatment inhibited subsequent viral genome replication in Huh7.5 cell line, chosen for high viral replication due to a RIG-I mutation suppressing innate antiviral signaling [119]), suggesting potential functions beyond cell entry. Extracellular HSPGs are relatively proline-rich and viral polyproteins ORF1a and ORF1ab, as well as spike protein, are likewise proline-rich even compared with proline-rich cellular collagen. The inhibition of ProRS activity by halofuginone suggests that infection-directed synthesis of proline-rich viral and cellular proteins might be an attractive target for design of novel antiviral therapeutics. Consistent with this rationale, two other ProRS inhibitors, i.e. ProSA (a non-hydrolyzable prolyl-AMP analog) and halofuginol, also inhibited infection by SARS-CoV-2 [117].

## An unconventional multi-aaRS complex directed by COVID-19 cues

mRNA termini are critical for agonist-driven, post-transcriptional regulons, in which functionally related mRNAs are co-regulated by specific RNA-binding proteins targeting similar sequence or structural elements [120]. The Gamma-interferon-Activated Inhibitor of Translation (GAIT) RNA element in a family of inflammation-related human mRNAs [48,121–124] is targeted by an inducible, heterotetrameric GAIT complex comprised of EPRS1, the direct RNA-interacting constituent, as well as ribosomal protein L13a, heterogenous ribonucleoprotein Q or NSAP1, and GAPDH; the system is a classical archetype of a post-transcriptional regulon. SARS-CoV-2 transcription generates an ensemble of nested 3′-co-terminal subgenomic RNAs (sgRNAs) that contain 5′-leader and 3′-end sequences identical with each other, and to the genomic RNA (gRNA). Upon interrogation of the 3′-end of SARS-CoV-2 for GAIT element-like RNA elements, a novel 39 nt element present in all viral RNAs was described [125]. This *cis*-element is structurally homologous to the pig alphacoronaviral TGEV (transmissible gastroenteritis virus) GAIT-like element [48]. The RNA sequence is conserved in SARS-CoV-1 and other viruses of the subgenus Sarbecovirus suggesting an invariant function. Insulin and IFN-γ, agents associated with COVID-19 severity and outcome, increase SARS-CoV-2 sgRNA expression and translation contingent upon an intact *cis*-element; disruption of the proposed secondary structure led to loss of agonist-mediated induction. Maximal reporter activation was observed following co-treatment with spike subunit S1 (the RBD) and IFN-γ in lung and colon cell lines expressing the ACE2 receptor. The newly discovered *cis*-element was termed Sarbecoviral Pan-End Activating RNA (SPEAR) element.

Beyond its influence on sgRNA reporter expression, an intact SPEAR element was required for maximal −1 programmed ribosomal frameshifting (−1 PRF) efficiency in frameshift-assay reporters bearing SARS-CoV-2 genomic termini, thereby expanding the role of SPEAR. The SPEAR element is rooted in the structurally ‘fluid' 3′-end hypervariable region (HVR) in subgenus Sarbecovirus — a region of weak sequence conservation among different Betacoronavirus subgenera, and therefore this sequence is absent from the subgenera Embecovirus, Nobecovirus and Merbecovirus, while in the monotypic subgenus Hibecovirus, only the sequence corresponding to the SPEAR proximal stem is conserved [125].

GAIT and GAIT-like TGEV RNA elements bind EPRS1, an aaRS that resides in the multi-tRNA synthetase complex (MSC) [41,42,126]. The SPEAR interacts with EPRS1 and NSAP1, but not with RPL13a or GAPDH, in IFN-γ-programmed U937 monocytic cell extracts [125]. Disruption of the SPEAR element by mutation prevents binding of EPRS1, but not NSAP1, further distinguishing the SPEAR-binding and GAIT complexes. More importantly, SPEAR disruption inhibits sgRNA expression and further distinguishes SPEAR function from GAIT function as the former activates and the latter inhibits expression. EPRS1 requirement for SPEAR activation was demonstrated by genetic perturbation in adipocytes and knockdown in a lung cell line. Unexpectedly, a subset of MSC constituents in addition to EPRS1, namely MARS1, KARS1, and RARS1, interacts with SPEAR in extracts from an IFN-γ- or insulin-treated lung cell line. UV cross-linking revealed both EPRS1 and KARS1 directly interacted with the SPEAR element; EPRS1 binding was mediated by the linker domain — the region that also binds the GAIT element. Interaction of EPRS1 with SARS-CoV-2 sgRNAs as well as gRNA was confirmed in IFN-γ stimulated SARS-CoV-2 replicon. Following cell induction with IFN-γ or spike S1, or both, the four SPEAR-binding aaRSs mobilize to form an extra-MSC, ∼500 kDa *t*etra-*a*minoacyl-tRNA synthetase *s*arbecoviral *R*NA-*i*nteracting (TASRI) complex — the second largest known human aaRS complex, after the MSC. The agonists induce relocalization of the TASRI complex to the endoplasmic reticulum (ER), a primary source of the double-membrane organelles supporting SARS-CoV-2 genome replication [127]. Importantly, cell-penetrating peptide-phosphorodiamidate morpholine oligonucleotide (PPMO) conjugates antisense to the SPEAR element block EPRS1 (and TASRI complex) binding, exhibit nearly 1.5-log reduction in SARS-CoV-2 titers, reduce viral protein levels in infected cells, attenuated growth kinetics in a SARS-CoV-2 EGFP reporter virus and effectively reduce viral genomic and subgenomic RNAs, indicating an important role of the interaction of the TASRI complex with SPEAR-element in SARS-CoV-2, and its potential as a therapeutic target [125].

Within the MSC, EPRS1 and MARS1 reside in a subcomplex joined by interacting GST-like domains; according to a current model RARS1 and KARS1 reside in a disconnected MSC region, complexed with AIMP2 and QARS1 [126]. Agonist-induced release of EPRS1 and KARS1 from the cytoplasmic MSC has been reported [45,49]. Formation of the TASRI complex is not understood and might not be a one-step dissociation from the cytoplasmic MSC, but rather an orchestrated, multi-step dissociation-association process. Moreover, parts of the TASRI complex may be derived from newly generated, free cytoplasmic pools. Translational control of SARS-CoV-2 sgRNAs that is SPEAR-dependent and regulated by a host aaRS complex is a unique example of a cellular complex-stimulated regulatory function of an RNA element in the SARS-CoV-2 3′-end.

**Figure 2.**
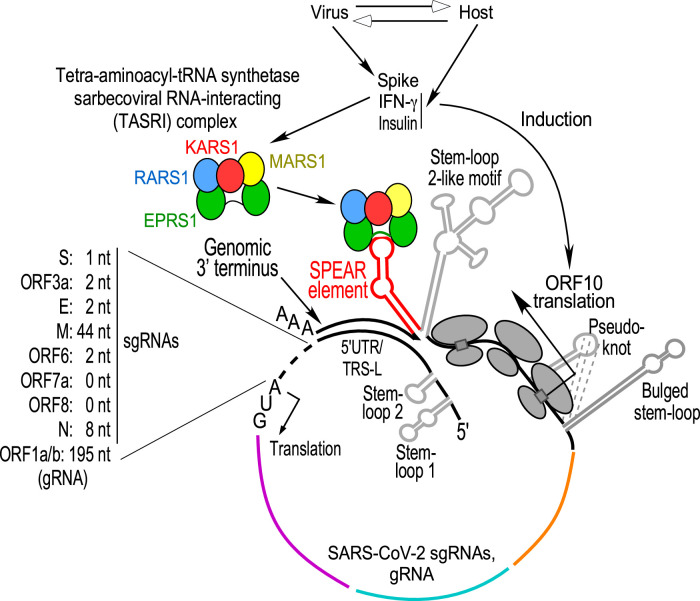
An integrated model of SARS-CoV-2 ORF10, SPEAR element, and virus RNA cyclization [125]. RNAs are cyclized by base-pairing of the 3′-end genomic terminus immediately downstream of the SPEAR element, with 16 complementary nucleotides of the TRS-L (transcription regulatory sequence-L) or 21 nt of the 5′UTR [128]. The start codon lies 0–2 nt downstream of this base-paired region in most sgRNAs, except in N (8 nt) and M (44 nt); in gRNA, the ORF1a/b start codon lies further downstream, with intervening 5′UTR stem–loop structures (not shown). Cell activation by SARS-CoV-2 spike, IFN-γ, or insulin induces ORF10 translation and formation of the SPEAR element-binding TASRI (tetra-aminoacyl-tRNA synthetase sarbecoviral RNA-interacting) complex.

The SPEAR element resides in the structurally ambiguous HVR. Internal initiation at ORF10, a 3′-end co-terminal feature newly acquired in SARS-CoV-2, plays a critical role in SPEAR-mediated induction of sgRNA expression [125], Mechanistically, this finding suggests a potential role of translating ribosomes in activating the SPEAR element by TASRI complex-assisted RNA refolding. We propose a framework to decode structure–function relationships of SARS-CoV-2 3′-end regulatory regions, where (i) functional translation of *ORF10* forms a SPEAR-permissive 3′ end, (ii) an agonist-inducible TASRI complex directs SPEAR element formation in the structurally fluid HVR, (iii) the SPEAR-adjacent genomic terminus base-pairs with the 5′UTR in gRNA/5′TRS-L (transcription regulatory sequence of leader) in sgRNAs [128], and (iv) sgRNA or gRNA circularization places the SPEAR element proximate to the start codon to regulate translation and PRF [125] (Figure 2). Additionally, the SPEAR element is near the virus 3′-end triple-helix junction necessary for recognition by the replication–transcription complex (RTC), possibly bringing the TASRI complex in proximity to the viral replication machinery as well.

SPEAR element activation by IFN-γ, insulin, and spike in multiple cell types suggests pathophysiologic significance. EPRS1 binding to the SPEAR element was markedly increased in lysates from epididymal white adipose tissue from fat-fed obese mice compared with normal mice. Adipose tissues are virus reservoirs, as well as sources of inflammatory adipokines, contributing to COVID-19 severity in obese patients [129,130]. The relative risk of death in severely obese COVID-19 patients is ∼4.2-fold higher compared with non-obese patients [131]. Likewise, visceral fat area is associated with an increased need for treatment in intensive care units and for mechanical ventilation [132]. Elevated EPRS1 binding to the SPEAR element in adipose tissue from obese mice suggests a mechanism underlying risk of severe COVID-19 in obese patients [125]. Similarly, insulin stimulates EPRS1 binding to SPEAR in differentiated adipocytes, possibly illuminating the clinical observation that insulin treatment, like obesity, is associated with increased mortality in COVID-19 patients [133]. Importantly, substantial weight loss following bariatric surgery in obese patients is associated with improved outcomes of COVID-19 infection, suggesting a causal relationship, and that obesity is a modifiable risk factor for COVID-19 severity [134]. IFN-γ, like other inflammatory cytokines, exhibits potent antiviral activity. However, uncontrolled levels of circulating cytokines and immune cell hyperactivation are characteristic of the ‘cytokine storm', a life-threatening, systemic inflammatory syndrome, and a principal contributor to COVID-19 pathogenesis [135]. The circulating level of IFN-γ, unique among cytokines measured, is higher in COVID-19 patients that succumbed, compared with survivors [136]. Treatment of mice with IFN-γ, in combination with TNF-α, induces a cytokine shock that mirrors the tissue damage of COVID-19, and neutralizing antibodies targeting these cytokines protect mice from mortality following SARS-CoV-2 infection [137]. IFN-γ induces expression of the ACE2 receptor of SARS-CoV-2 in enterocytes and promotes virus production in infected cells [138]. IFN-γ-induced activation of the SPEAR-binding complex might provide an additional pathogenic mechanism [125]. In addition to host agonists, SARS-CoV-2 spike also activates the SPEAR-binding complex. Spike engages the cellular ACE2 receptor for virus entry and elicits cell signal transduction pathways that promote pulmonary and cardiovascular complications [139,140]. Additionally, spike can drive viral infection and pathogenesis via non-ACE2 interactions in cells with low ACE2 receptor levels [141,142]. The S1 subunit of spike, containing N-terminal and receptor-binding domains, induces EPRS1 incorporation into a complex with three other MSC-constituent aaRSs, and binding to the SPEAR element via the EPRS1 non-catalytic linker domain [125]. Together, the results show that SPEAR is a novel, pan-sgRNA translation–activation element that, along with a newly elucidated host-derived TASRI complex, defines a SARS-CoV-2 post-transcriptional regulon.

## Future directions

Accelerating disruptive activities of mankind have led to habitat destruction of many organisms, increasing future likelihood of zoonotic spillover of viruses. Lessons learned from COVID-19 will be important for navigating a future pandemic from known or as-yet unknown viruses, including sarbecoviruses [60,143,144]. Near-term, elucidation of the role of aaRSs in translation, replication, infection, and pathogenesis of SARS-CoV-2 variants might explain differences in their pathogenesis. The knowledge gathered in aaRS biology as mediators of virus infection will facilitate design of new antiviral therapeutic strategies.

## Perspectives

- Aminoacyl-tRNA synthetases have canonical, post-canonical, and non-canonical functions in cellular homeostasis and in response to environmental stimuli.
- The repertoire of non-canonical functions of aminoacyl-tRNA synthetases extend to being host modulators of virus infection.
- Recent elucidation of molecular aspects in SARS-CoV-2 infection have expanded the galaxy of interactions exhibited by these proteins, as also exemplified by a previously unrecognized TASRI complex comprised of four aminoacyl-tRNA synthetases.

## Open Access

Open access for this article was enabled by the participation of Cleveland Clinic in an all-inclusive *Read & Publish* agreement with Portland Press and the Biochemical Society under a transformative agreement with Individual.

## Abbreviations

aa: amino acid
aaRS: aminoacyl-tRNA synthetase
ACE2: angiotensin converting enzyme 2
AIMP: aminoacyl-tRNA synthetase complex-interacting multifunctional protein
AMP: adenosine monophosphate
ARDS: acute respiratory distress syndrome
ATP: adenosine triphosphate
BALF: bronchoalveolar lavage fluid
BMV: brome mosaic virus
COVID-19: coronaviral disease-19
CRISPR: clustered regularly interspaced short palindromic repeats
CysSSH: cysteine hydropersulfide
cyto-aaRS: cytosolic aminoacyl-tRNA synthetase
GAIT: gamma-interferon activated inhibitor of translation
gRNA: genomic RNA
GST: glutathione S transferase
H1N1 influenza A virus subtype-hemagglutinin 1: neuraminidase 1
hGatCAB: human glutamyl-tRNA(Gln) amidotransferase subunits C-A-B
HIV: human immunodeficiency virus
HS: heparan sulfate
HSPG: heparan sulfate proteoglycan
HSV: herpes simplex virus
HVR: hypervariable region
IFN: interferon
MAVS: mitochondrial antiviral-signaling protein
MERS-CoV: middle east respiratory syndrome coronavirus
mRNA: messenger RNA
MSC: multi-aminoacyl tRNA synthetase complex
mt-aaRS: mitochondrial aminoacyl-tRNA synthetase
NSP: non-structural protein
ORF: open reading frame
PCBP2: poly(rC)-binding protein 2
RBD: receptor-binding domain
RSV: respiratory syncytial virus
RTC: replication–transcription complex
S1: spike subunit 1
SARS-CoV-2: severe acute respiratory syndrome coronavirus 2
sgRNA: subgenomic RNA
SPEAR: sarbecoviral pan-end activating RNA
TASRI complex: tetra-aminoacyl-tRNA synthetase sarbecoviral RNA-interacting complex
TGEV: transmissible gastroenteritis coronavirus
TLE: tRNA-like element
TLS: tRNA-like structures
TMPRSS2: transmembrane serine protease 2
TNF: tumor necrosis factor
tRNA: transfer RNA
TRS-L: transcription regulatory sequence of leader
UTR: untranslated region
VSV: vesicular stomatitis virus
